# Conventional endovascular treatment and flow diverter for unruptured small- and medium-sized paraophthalmic segment aneurysms

**DOI:** 10.3389/fneur.2025.1648848

**Published:** 2025-11-04

**Authors:** Kefeng Liu, Yong Sun, Fang Liu, Xin Zhang, Aimin Li

**Affiliations:** ^1^Digital Intelligence Neural Research Laboratory, Department of Neurosurgery, The Second People’s Hospital of Changzhou, The Third Affiliated Hospital of Nanjing Medical University, Changzhou, Jiangsu, China; ^2^Department of Neurosurgery, Lianyungang Clinical College of Nanjing Medical University, Lianyungang, Jiangsu, China; ^3^Department of Neurosurgery, Jinling School of Clinical Medicine, Jinling Hospital, Nanjing Medical University, Nanjing, China

**Keywords:** paraophthalmic segment aneurysm, endovascular treatment, flow diverter, efficacy and safety, propensity score matching

## Abstract

**Objective:**

The efficacy and safety of flow diverters (FD) compared to conventional endovascular treatment (CET) for treating small- and medium-sized intracranial aneurysms remain unclear. This study aimed to compare the efficacy and safety of FD and CET in common small- and medium-sized paraophthalmic segment aneurysms (PSAs) in clinical practice, with the expectation of providing a basis for clinical treatment decisions for such aneurysms.

**Methods:**

This multicenter, retrospective cohort study included consecutive patients with unruptured small- and medium-sized (≤10 mm) PSAs treated at three neurosurgical centers between January 2018 and December 2023. Patients were assigned to the CET or FD group. The primary efficacy endpoint was the rate of complete aneurysm occlusion. The safety endpoints included perioperative and postoperative complications. Propensity score matching (PSM) was used to verify the stability of the results. Subgroup analyses were conducted to identify the factors influencing clinical outcomes.

**Results:**

A total of 688 PSAs in 601 patients were analyzed, with 595 cases treated with CET and 93 cases treated with FD. The mean follow-up duration for all cases was 12.6 ± 12.9 months. The complete occlusion rate was significantly higher in the CET group than in the FD group (98.2% versus 66.7%, *p* < 0.001) at the last follow-up. However, the cumulative incidence of aneurysm occlusion increased over time in both groups, with no significant difference between the groups (log-rank test, *p* = 0.261). Compared with the CET group, the adjusted hazard ratio (HR) for complete occlusion in the FD group was 0.632 (95% confidence interval [CI]: 0.307–1.299; *p* = 0.212). The FD group had a higher overall complication rate (12.0% versus 1.1%; *p* = 0.007); nevertheless, these complications did not significantly affect long-term functional outcomes. The findings remained robust after PSM. Subgroup analysis revealed that the efficacy advantage of CET was more prominent in older patients (≥ 65 years).

**Conclusion:**

The rates of complete occlusion for small- and medium-sized PSAs were not significantly different between CET and FD therapy. However, the procedural safety profile of FD requires careful consideration. CET demonstrated a more pronounced therapeutic benefit in elderly patients than in younger patients.

## Introduction

According to the published literature, the global prevalence of unruptured intracranial aneurysms in individuals around the age of 50 years is approximately 3% (1). Aneurysm rupture can cause severe disability or death, imposing a substantial psychological and economic burden on patients and their families. Paraophthalmic segment aneurysms (PSAs) originate from the distal segment of the internal carotid artery beyond the cavernous sinus and extend to the origin of the posterior communicating artery, encompassing both the clinoid and ophthalmic segments (2, 3). Clinically, aneurysms in this location are relatively common, accounting for 1.3–10% of intradural aneurysms (4–6). Among aneurysms incidentally discovered during physical examinations, PSAs constitute a significant proportion, accounting for approximately 3%, and are predominantly small to medium in size (5).

Currently, the primary endovascular treatment approaches for PSAs include conventional endovascular treatment (CET) and flow diverter (FD) therapy (7, 8). Traditional methods, including coil embolization, stent-assisted coiling, and balloon-assisted coiling, have been widely used. However, they have certain limitations in achieving complete and permanent aneurysm occlusion, particularly for complex, large, or giant aneurysms that are at a high risk of recurrence (9). Meta-analyses have indicated that the recurrence rate after CET can be as high as 14% (10), with 10% of cases requiring retreatment (11).

To address these limitations, FD has recently been increasingly adopted for treating intracranial aneurysms (12). FD offers a novel therapeutic approach by reconstructing the parent artery and altering the hemodynamics inside and outside the aneurysm sac (13). Several studies have demonstrated that FD treatment offers a higher rate of complete aneurysm occlusion than CET, more than double in some reports (14). However, the incidence of procedure-related complications is higher with FD than with CET (15).

The “Chinese Guidelines for Flow Diverter Treatment of Intracranial Aneurysms (2022)” highlights the high efficacy and safety of FD therapy for unruptured large and giant internal carotid artery aneurysms, making it the conventional indication for wide-necked, large, and giant aneurysms (16). However, these guidelines do not explicitly recommend FD over CET for small- or medium-sized internal carotid artery aneurysms. Three-year follow-up data from the PREMIER study revealed that FD achieved durable and high occlusion rates in treating wide-necked small- and medium-sized aneurysms, with a low incidence of neurological complications (17, 18). However, some studies have found that although FD achieves higher complete occlusion rates than traditional stent-assisted coiling in small intracranial aneurysms, this advantage is accompanied by a relatively increased risk of adverse events (19). Therefore, the optimal treatment strategy for small- and medium-sized PSAs remains controversial (20).

This study aimed to compare the efficacy and safety of FD and CET in treating commonly encountered, predominantly located, small- and medium-sized PSAs and to provide a more robust evidence base for clinical decision-making in managing these aneurysms.

In summary, this multicenter, propensity-score–matched cohort study uniquely compares the real-world efficacy and safety of flow diverters and conventional endovascular treatments for unruptured small- and medium-sized PSAs. By addressing current evidence gaps concerning treatment selection for this specific aneurysm subtype, our research provides comprehensive complication profiles, long-term occlusion trends, and robust subgroup evidence. These novel and clinically relevant findings directly inform personalized therapeutic strategies and may critically impact future guideline recommendations for the management of small- and medium-sized PSAs.

## Materials and methods

### Study design and ethical statement

This multicenter retrospective cohort study aimed to compare the efficacy and safety of CET and FD therapy for small- and medium-sized (maximum diameter ≤ 10 mm) unruptured PSAs. All patients were treated at the neurosurgery departments of three major hospitals in Jiangsu Province, China: Eastern Theater Command General Hospital, Lianyungang First People’s Hospital, and Changzhou Second People’s Hospital. Patients treated consecutively between January 2018 and December 2023 were included.

All treatment strategies were determined by neurointerventional teams (consisting of eight operators, each with at least 5 years of experience) based on individualized assessments. Treatment selection followed the guidelines and multi-center consensus according to the following principles: (1) FD was the first-line option for multiple ipsilateral internal carotid artery aneurysms; (2) CET was prioritized for narrow-neck or regular saccular aneurysms, whereas FD was preferred when coil stability was poor, the neck was extremely wide, or flow remodeling was required; (3) CET was favored in patients with subtherapeutic platelet inhibition rates on antiplatelet therapy; (4) FD was preferred when the ophthalmic artery originated from the aneurysm dome; and (5) the final plan required consensus from at least two senior operators and informed consent from the patient.

This study followed the principles of the Helsinki Declaration and was approved by the ethics committees of all three participating centers. The requirement of written informed consent was waived owing to the retrospective nature of the study and the use of anonymized data.

### Inclusion and exclusion criteria

**Figure 1:**
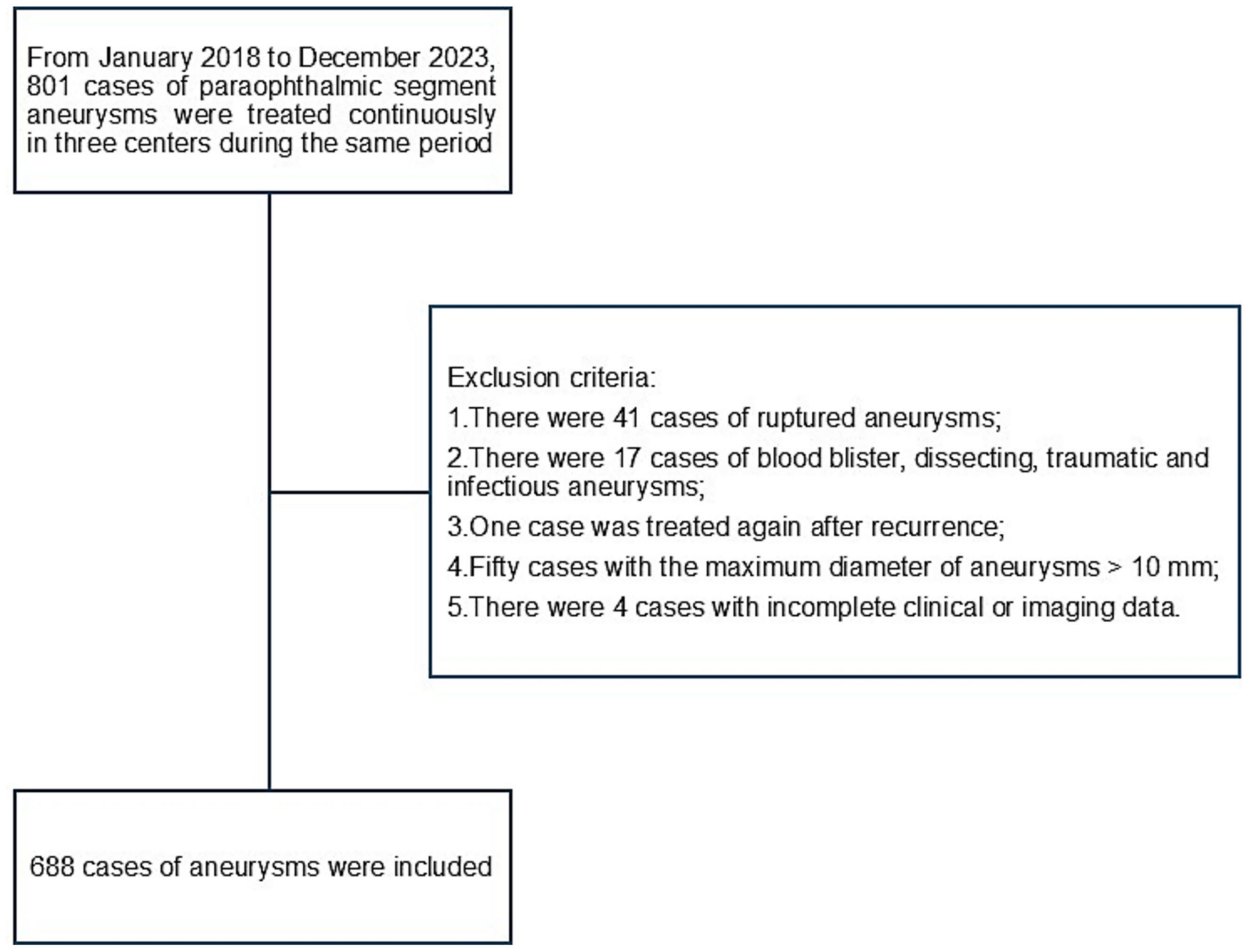
Patient’s flowchart.

The inclusion criteria for this study were as follows: (1) PSA confirmed using digital subtraction angiography (DSA) and (2) age ≥18 years. The exclusion criteria were as follows: (1) Ruptured aneurysms; (2) blister-like, dissecting, traumatic, or infectious aneurysms; (3) prior aneurysm clipping or endovascular retreatment; (4) aneurysm maximum diameter of > 10 mm; (5) incomplete clinical or imaging data. A detailed patient selection flowchart is presented in Figure 1.

### Perioperative management and endovascular procedures

Patients were assigned to CET or FD. CET comprises simple coiling for narrow-neck aneurysms and stent-assisted coiling for wide-neck lesions (21). FD is performed with Pipeline (Medtronic), Tubridge (MicroPort), or Surpass (Stryker), with adjunctive coiling when indicated. FD sizing was based on three-dimensional angiographic measurements of parent artery diameters and planned landing zones; devices were delivered via a triaxial system with fluoroscopic confirmation of deployment and wall apposition, and balloon angioplasty was used for incomplete expansion or malapposition. For patients with bilateral aneurysms, the contralateral lesion is typically treated within 1–3 months after the index procedure.

Antiplatelet management was standardized: no antiplatelet therapy was used for simple coiling; for stent-assisted coiling or FD, dual antiplatelet therapy (DAPT; aspirin 100 mg/day plus clopidogrel 75 mg/day) was initiated 5–7 days preoperatively and adjusted by thromboelastography to a 30–90% platelet inhibition target (clopidogrel 150 mg/day if <30%; switch to ticagrelor 60 mg twice daily if still suboptimal). Intraoperatively, systemic heparinization maintained the activated clotting time (ACT) at 2–3 times the baseline. Postoperatively, DAPT is continued for 3–6 months, followed by long-term single-agent therapy (22, 23).

### Data collection and parameter definitions

Demographic data, medical history, aneurysm morphological parameters, and procedural details were extracted from the electronic medical record system. DSA imaging data were obtained using an Artis zee biplane angiography system (VC14; Siemens, Munich, Germany). Two independent neurosurgeons, blinded to the clinical details, measured the aneurysm parameters, and the mean of their measurements was used for analysis. The maximum aneurysm diameter was defined as the greatest distance between any two points on the aneurysm sac; neck diameter as the mean width of the neck plane; aspect ratio (AR) as the aneurysm height perpendicular to the neck plane divided by the neck diameter; size ratio (SR) as the maximum aneurysm diameter divided by the diameter of the parent artery; bottle neck ratio (BNR) as the maximum diameter divided by the neck diameter.

### Clinical and imaging follow-up

Perioperative and postoperative complications, as well as clinical symptoms and signs, were recorded using an electronic medical record system. Imaging follow-ups were performed at 6 months, 1 year, 2 years, and 3 years postoperatively using DSA or magnetic resonance angiography (MRA) (18). If complete thrombosis of the aneurysm was achieved, annual MRA follow-up was continued. If the aneurysm displayed no change or incomplete thrombosis, repeat endovascular treatment was considered based on the clinical scenario. Clinical follow-up was conducted in the outpatient clinic or by telephone, documenting the patients’ conditions, modified Rankin Scale (mRS) scores, ocular symptoms, and any possible complications that may have occurred.

### Outcome measures

The primary efficacy outcomes included the rate of complete aneurysm occlusion, mRS score, and improvement in ocular symptoms. A good prognosis was defined as an mRS score of 0–2 at the final follow-up. For aneurysms treated with CET, occlusion status was evaluated using the Raymond–Roy occlusion classification on DSA, with grade I indicating complete occlusion and grades II and III indicating incomplete occlusion. The O’Kelly–Marotta grading scale was used for aneurysms treated with FD: complete filling (A), partial filling (B), neck remnant (C), and no filling (D). Grades A, B, and C were considered incomplete occlusions, whereas grade D indicated complete occlusion. On MRA follow-up, the absence of contrast filling in both the aneurysm sac and neck was defined as complete occlusion.

The safety endpoints included procedure-related complications, such as intraoperative hemorrhage, thrombosis, air embolism, and stent malapposition. Postoperatively, until the last follow-up, additional procedure-related complications were documented, including delayed hemorrhage, ischemia, cranial nerve dysfunction, stent migration, and in-stent stenosis (parent artery stenosis of > 50%). All adverse events, including procedure-related mortality, that occurred during or after the procedure were included in the safety analysis.

### Statistical analysis

Continuous variables are expressed as mean ± standard deviation (SD) or median (interquartile range), as appropriate. Independent sample t-tests were used for normally distributed variables, and the Mann–Whitney *U* test was applied for non-normally distributed variables. Categorical variables are presented as frequencies (percentages). Group differences were assessed using the *χ*^2^ test or Fisher’s exact test, as appropriate. Propensity score matching (PSM) was employed to control for confounding bias between CET and FD groups. Matching variables included age, gender, body mass index, aneurysm location, history of hyperlipidemia, hypertension, diabetes mellitus, coronary heart disease, cerebral hemorrhage, cerebral infarction, subarachnoid hemorrhage, current smoking status, preoperative mRS score (0–2), and maximum aneurysm diameter. The caliper was set at 0.01, and 1:1 nearest-neighbor matching was performed.

Subgroup analyses were conducted using interaction testing (likelihood ratio test) to evaluate the influence of different baseline characteristics on aneurysm occlusion and complications. The results were visualized using forest plots. The Kaplan–Meier (KM) method was used to analyze the cumulative incidence of complete aneurysm occlusion for the two treatment modalities, and the KM curves were plotted. The log-rank test was used to compare the time-to-event distributions between groups. To compare the time to aneurysm occlusion between the FD and CET groups, we fitted Cox proportional hazards models. The multivariable model adjusted for age, sex, body mass index, hyperlipidemia, hypertension, diabetes mellitus, coronary heart disease, and maximum aneurysm diameter. For variables with a missing rate of < 10%, multiple imputations were performed to complete the data. All statistical analyses were performed using R software (version 4.3.3, R Foundation) and EmpowerStats software (X&Y Solutions). All tests were two-sided, and *p*-values < 0.05 were considered statistically significant.

## Results

### Baseline characteristics

After screening, 688 PSAs from 601 patients were included in this study. Among these, 524 (76.2%) were small aneurysms with diameters < 5 mm, and 164 (23.8%) were medium-sized aneurysms with diameters of 5–10 mm. CET was performed in 595 cases, while FD treatment was applied in 93 cases (Table 1).

**Table 1:** Baseline characteristics of patients treated with CET versus FD.

Characteristics	CET (*n* = 595)	FD (*n* = 93)	*p*-value
Age (years, mean ± SD)	56.7 ± 11.3	55.7 ± 10.6	0.453
Gender, *n* (%)			0.698
Male	155 (26.1%)	26 (27.9%)	
Female	440 (73.9%)	67 (72.1%)	
Body mass index, mean ± SD	24.1 ± 3.0	24.5 ± 3.4	0.460
Aneurysm location, *n* (%)			0.096
Left	316 (53.1%)	58 (62.3%)	
Right	279 (46.8%)	35 (37.6%)	
History of hyperlipidemia, *n* (%)	43 (7.2%)	7 (7.5%)	0.917
History of hypertension, *n* (%)	229 (38.5%)	31 (33.3%)	0.340
History of diabetes mellitus, *n* (%)	64 (10.7%)	12 (12.9%)	0.539
History of cerebral hemorrhage, *n* (%)	12 (2.0%)	2 (2.1%)	0.932
History of cerebral infarction, *n* (%)	66 (11.1%)	6 (6.5%)	0.174
History of coronary heart disease, *n* (%)	13 (2.2%)	2 (2.2%)	0.983
History of subarachnoid hemorrhage, *n* (%)	11 (1.9%)	1 (1.1%)	0.596
Current smoker, *n* (%)	57 (9.6%)	3 (3.2%)	0.043
Preoperative mRS Score, *n* (%)			0.834
Score 0	580 (97.5%)	92 (98.9%)	
Score 1	12 (2.0%)	1 (1.1%)	
Score 2	1 (0.2%)	0 (0.0%)	
Score 3	2 (0.3%)	0 (0.0%)	
Preoperative cranial nerve palsy, *n* (%)	6 (1.0%)	1 (1.1%)	1.000
Preoperative visual impairment, *n* (%)	5 (0.8%)	1 (1.1%)	0.583
Clopidogrel resistance, *n* (%)	151 (25.6%)	23 (24.7%)	0.866
Number of aneurysms, *n* (%)			<0.001
Single	390 (65.6%)	30 (32.3%)	
Two	160 (26.9%)	31 (33.3%)	
Three	39 (6.6%)	17 (18.3%)	
Four	5 (0.8%)	15 (16.1%)	
Five	1 (0.2%)	0 (0.0%)	
Multiple aneurysms, *n* (%)	205 (34.5%)	63 (67.7%)	<0.001
Irregular aneurysm shape, *n* (%)	532 (95.7%)	83 (90.2%)	0.027
Aneurysms with daughter sac, *n* (%)	71 (12.8%)	12 (13.0%)	0.937
Maximal aneurysm diameter (mm, mean ± SD)	4.1 ± 1.7	3.7 ± 2.1	0.031
Aneurysm neck diameter (mm, mean ± SD)	3.32 ± 1.01	3.26 ± 1.19	0.112
Size ratio (SR, mean ± SD)	0.9 ± 0.46	0.86 ± 0.57	< 0.001
Aspect ratio (AR, mean ± SD)	1.07 ± 0.55	0.94 ± 0.42	0.009
Bottle neck ratio (BNR, mean ± SD)	1.18 ± 0.52	1.07 ± 0.30	0.042

SD, standard deviation; CET, conventional endovascular treatment; FD, flow diverters.

The two groups did not differ statistically significantly regarding demographics or comorbidities, including age (56.7 ± 11.3 versus 55.7 ± 10.6 years; *p* = 0.453), gender (male, 26.1% versus 27.9%; *p* = 0.698), body mass index (24.1 ± 3.0 versus 24.5 ± 3.4; *p* = 0.460), or aneurysm laterality (left, 53.1% versus 62.3%; *p* = 0.096). The prevalence of hyperlipidemia (7.2% versus 7.5%; *p* = 0.917), hypertension (38.5% versus 33.3%; *p* = 0.340), or diabetes mellitus (10.7% versus 12.9%; *p* = 0.539) did not differ significantly. The smoking rate was significantly higher in the CET group than in the FD group (9.6% versus 3.2%; *p* = 0.043).

Regarding aneurysm-related characteristics, the proportion of patients with multiple aneurysms was significantly higher in the FD group (67.7% versus 34.5%; *p* < 0.001), and the distribution of the number of aneurysms was significantly different (single aneurysm: 32.3% versus 65.6%; *p* < 0.001). In addition, the mean aneurysm diameter (3.7 ± 2.1 mm versus 4.1 ± 1.7 mm; *p* = 0.031), SR (0.86 ± 0.57 versus 0.9 ± 0.46; *p* < 0.001), AR (0.94 ± 0.42 versus 1.07 ± 0.55; *p* = 0.009), and BNR (1.07 ± 0.30 versus 1.18 ± 0.52; *p* = 0.042) were significantly lower in the FD group than in the CET group (Table 1).

The most commonly used stent in the CET group was Enterprise (86.6%), followed by Atlas (3.5%), LVIS (2.2%), Solitaire (1.3%), and other stents (6.4%). The most commonly used stent in the FD group was Tubridge (40.9%), followed by Pipeline (37.6%), Surpass (6.5%), and other devices (15.0%).

### Clinical efficacy

Of the 688 aneurysms, 595 (86.5%) were treated with CET, and 93 (13.5%) were treated with FD. The overall follow-up rate was 64.5% (444/688), with 62.4% (371 cases) in the CET group and 78.5% (73 cases) in the FD group (*p* = 0.002). The mean follow-up duration for all cases was 12.6 ± 12.9 months (CET, 13.3 ± 13.9 months; FD, 9.4 ± 4.3 months), with no statistically significant difference between the groups (*p* = 0.759).

At the last follow-up, the aneurysm occlusion rate was significantly higher in the CET group than in the FD group (96.8% versus 65.8%; *p* < 0.001). The rate of good clinical outcomes (mRS score, 0–2) did not differ significantly between CET and FD groups (98.8% versus 97.9%; *p* = 0.349; Table 2). One patient in the CET group required retreatment due to aneurysm recanalization, but no aneurysm rupture was observed. Two procedure-related mortality occurred in the CET group (2/595, 0.3%): one patient died 2 weeks postoperatively from severe cardiopulmonary failure, and another died 1 month after surgery due to ruptured abdominal aortic aneurysm. No deaths occurred in the FD group (0/93, 0.0%) (Table 2).

**Table 2:** Comparison of clinical outcomes and safety between CET and FD for aneurysm treatment.

Outcomes	CET (*n* = 595)	FD (*n* = 93)	*P*-value
Follow-up duration (months, median [IQR])	7.1 (6.0–14.9)	6.90 (6.5–12.3)	0.759
Complete occlusion at last follow-up, *n* (%)	359 (96.8%)	48 (65.8%)	<0.001
Intraoperative complications, *n* (%)	5 (0.8%)	5 (5.4%)	<0.001
Intraoperative hemorrhage, *n* (%)	2 (0.3%)	0 (0.0%)	1.000
Intraoperative air embolism, *n* (%)	1 (0.2%)	0 (0.0%)	1.000
Intraoperative malapposition, *n* (%)	0 (0.0%)	4 (4.3%)	<0.001
Intraoperative thrombosis, *n* (%)	2 (0.3%)	1 (1.1%)	0.354
Postoperative complications, *n* (%)	29 (4.9%)	8 (8.6%)	0.138
Postoperative ischemia, *n* (%)	16 (2.7%)	4 (4.3%)	0.390
Postoperative hemorrhage, *n* (%)	3 (0.5%)	0 (0.0%)	1.000
Postoperative in-stent stenosis, *n* (%)	6 (1.0%)	7 (7.5%)	<0.001
Postoperative stent migration, *n* (%)	0 (0.0%)	0 (0.0%)	NA
Postoperative cranial nerve symptoms, *n* (%)	0 (0.0%)	1 (1.1%)	0.135
All adverse events, *n* (%)	31 (5.2%)	11 (11.8%)	0.013
Favorable outcome rate, *n* (%)	588 (98.8%)	91 (97.9%)	0.349
Mortality, *n* (%)	2 (0.3%)	0 (0.0%)	1.000

CET, conventional endovascular treatment; FD, flow diverters.

Analysis of the complete aneurysm occlusion rates at specific time points between the two treatment modalities revealed that the occlusion rate was significantly higher in the CET group than in the FD group (all *p* < 0.001) at each follow-up interval. This trend remained significant in the subgroup analysis that compared stent-assisted coil embolization and FD. Additionally, a comparison of simple coil embolization and stent-assisted coil embolization revealed that the occlusion rate in the simple coil group was 85.7% at short-term follow-up (≤6 months), which was lower than the 100% occlusion rate in the stent-assisted group; however, the difference was not statistically significant. However, the occlusion rate in the simple coil group significantly decreased (66.7% versus 94.2%; *p* = 0.187) at long-term follow-up (≥12 months). The overall occlusion rate in the simple coil group was 84.6% at the last follow-up, which was significantly lower than that in the stent-assisted coil group (97.2%; *p* = 0.012).

Further analysis revealed that the occlusion rate was significantly higher in the simple coil group than in the FD group (85.7% versus 27.8%; *p* = 0.021) during early follow-up (≤ 6 months). However, this advantage diminished over time, with the occlusion rate dropping from 100 to 69.6% at 6–12 months and from 66.7 to 68.0% at ≥12 months (Table 3).

**Table 3:** Follow-up results of aneurysm occlusion at different time periods and with different treatments.

Groups	≤6 months	6–12 months	≥12 months	Last follow-up
	(*n* = 132)	(*n* = 238)	(*n* = 131)	(*n* = 444)
CET versus FD				
CET, *n* (%)	113 (99.1%)	187 (97.4%)	99 (93.4%)	359 (96.8%)
FD, *n* (%)	5 (27.8%)	32 (69.6%)	17 (68.0%)	48 (65.8%)
*P*-value	<0.001	<0.001	<0.001	<0.001
Stent-assisted coiling versus FD				
Stent-assisted coiling, *n* (%)	107 (100.0%)	183 (97.3%)	8 (32.0%)	348 (97.2%)
FD, *n* (%)	5 (27.8%)	32 (69.6%)	17 (68.0%)	48 (65.8%)
*P*-value	<0.001	<0.001	<0.001	<0.001
Coiling versus stent-assisted coiling				
Coiling, *n* (%)	6 (85.7%)	4 (100.0%)	2 (66.7%)	11 (84.6%)
Stent-assisted coiling, *n* (%)	107 (100.0%)	183 (97.3%)	97 (94.2%)	348 (97.2%)
*P*-value	0.061	1.000	0.187	0.012
Coiling versus FD				
Coiling, *n* (%)	6 (85.7%)	4 (100.0%)	2 (66.7%)	11 (84.6%)
FD, *n* (%)	5 (27.8%)	32 (69.6%)	17 (68.0%)	48 (65.8%)
*P*-value	0.021	0.566	1.000	0.177

CET, conventional endovascular treatment; FD, flow diverters.

Over time, the occlusion rate in the CET group remained stable, whereas that in the FD group gradually increased from 27.8 to 68.0%. The stent-assisted coil group exhibited the highest occlusion rate (94.2%) at long-term follow-up (≥12 months), which was significantly higher than that of the simple coil and FD groups (both *p* < 0.001). Although the simple coil group displayed favorable early performance, its occlusion rate declined significantly from 100% at 6–12 months to 66.7% at ≥12 months.

**Figure 2:**
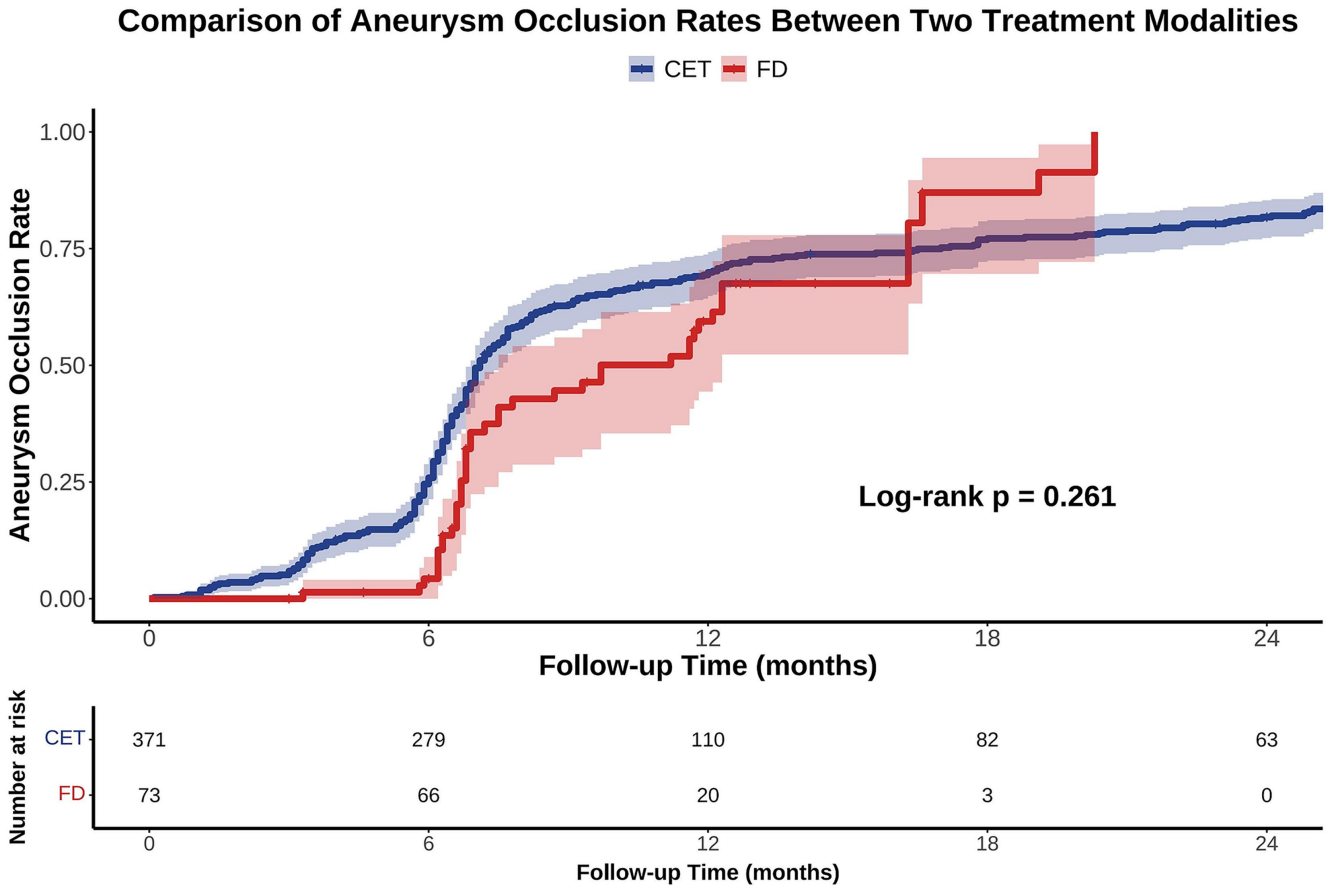
Kaplan–Meier (KM) analysis of aneurysm occlusion rates following CET versus FD treatment over 24 months. KM curves demonstrate the cumulative aneurysm occlusion rates for patients treated with CET (blue) and FD (red) during the 24-month follow-up period. Shaded areas represent the 95% CI. No significant difference was observed between the two groups (Log-rank *p* = 0.261). CET, conventional endovascular treatment; FD, flow diverters.

KM survival analysis revealed that the cumulative incidence of aneurysm occlusion increased over time in both the CET and FD groups, with median times to occlusion of 7.1 and 9.7 months, respectively; however, there was no significant difference between the two groups (log-rank test, *p* = 0.261; Figure 2). After adjusting for baseline characteristics in a multivariable Cox proportional hazards model, the hazard ratio (HR) for complete occlusion in the FD group compared to that in the CET group was 0.632 (95% confidence interval [CI]: 0.307–1.299; *p* = 0.212).

### Validation after PSM

The PSM was performed to balance the baseline characteristics between the two groups (92 cases in each group; Table 4) and control for confounding factors. After matching, the aneurysm occlusion rate remained significantly higher (98.2% versus 66.7%; *p* < 0.001) in the CET group, while the overall incidence of adverse events was still greater (12.0% versus 1.1%; *p* = 0.007) in the FD group, primarily due to a higher rate of postoperative in-stent stenosis (7.6% versus 1.1%; *p* = 0.071) and procedure-related complications (5.4% versus 0.0%; *p* = 0.070; Table 5). The rate of favorable outcomes did not differ significantly between the two groups (mRS score 0–2; 98.9% versus 97.8%; *p* = 1.000). Although the FD group displayed a higher risk of complications, it did not significantly affect the functional prognosis.

**Table 4:** Comparison of baseline characteristics between traditional endovascular treatment and flow diverter for aneurysm patients after PSM.

Characteristics	CET (*N* = 92*)	FD (*N* = 92)	*P*-value
Age (years, mean ± SD)	56.6 ± 10.2	56.1 ± 10.3	0.730
Gender, *n* (%)			1.000
Male	26 (28.3)	25 (27.2)	
Female	66 (71.7)	67 (72.8)	
Body mass index, mean ± SD	24.4 ± 3.1	24.4 ± 3.3	0.932
Aneurysm location			0.548
Left, *n* (%)	52 (56.5)	57 (62)	
Right, *n* (%)	40 (43.5)	35 (38)	
History of hyperlipidemia, *n* (%)	6 (6.5)	6 (6.5)	1.000
History of hypertension, *n* (%)	32 (34.8)	31 (33.7)	1.000
History of diabetes mellitus, *n* (%)	12 (13)	12 (13)	1.000
History of coronary heart disease, *n* (%)	1 (1.1)	2 (2.2)	0.477
History of subarachnoid hemorrhage, *n* (%)	2 (2.2)	1 (1.1)	1.000
Current smoker, *n* (%)	3 (3.3)	3 (3.3)	1.000
History of cerebral hemorrhage, *n* (%)	2 (2.2)	2 (2.2)	1.000
History of cerebral infarction, *n* (%)	9 (9.8)	6 (6.5)	0.590
Preoperative mRS score (0–2), *n* (%)	92 (100)	92 (100)	NA
Multiple aneurysms, *n* (%)	61 (66.3)	62 (67.4)	1.000
Maximal aneurysm diameter (mm, mean ± SD)	3.8 ± 1.6	3.8 ± 2.1	0.835

*N = 92 indicates the number of aneurysms included in the final analysis (of the 93 aneurysms in the FD group, one was excluded due to lack of a suitable matching aneurysm). CET, conventional endovascular treatment; FD, flow diverters; SD, standard deviation; mRS, modified Rankin Scale; PSM, propensity score matching.

**Table 5:** Comparison of clinical efficacy and safety outcomes between CET and FD after PSM.

Outcomes	CET (*N* = 92)	FD (*N* = 92)	*P*-value
Intraoperative complications, *n* (%)	0 (0)	5 (5.4)	0.070
Intraoperative hemorrhage, *n* (%)	0 (0)	0 (0)	NA
Intraoperative air embolism, *n* (%)	0 (0)	0 (0)	NA
Intraoperative malapposition, *n* (%)	0 (0)	4 (4.3)	0.129
Intraoperative thrombosis, *n* (%)	0 (0)	1 (1.1)	1.000
Postoperative complications, *n* (%)	1 (1.1)	8 (8.7)	0.040
Postoperative ischemia, *n* (%)	1 (1.1)	4 (4.3)	0.365
Postoperative hemorrhage, *n* (%)	0 (0)	0 (0)	NA
Postoperative in-stent stenosis, *n* (%)	1 (1.1)	7 (7.6)	0.071
Postoperative stent migration, *n* (%)	0 (0)	0 (0)	NA
Postoperative cranial nerve symptoms, *n* (%)	0 (0)	1 (1.1)	1.000
All adverse events, *n* (%)	1 (1.1)	11 (12)	0.007
Complete occlusion at last follow-up, *n* (%)	54 (98.2)	48 (66.7)	< 0.001
Favorable outcome rate, *n* (%)	91 (98.9)	90 (97.8)	1.000
Mortality, *n* (%)	1 (1.1)	0 (0)	1.000

PSM, propensity score matching; CET, conventional endovascular treatment; FD, flow diverters.

### Subgroup analysis and interaction effects

The benefit of CET was particularly pronounced (odds ratio [OR] = 0.01; 95% CI: 0.00–0.08; *p* < 0.001) in patients aged ≥ 65 years, whereas the OR increased to 0.10 (95% CI: 0.04–0.24; *p* < 0.001) in patients aged < 65 years. The interaction effect of age stratification was statistically significant (*P* for interaction = 0.032; Supplementary Figure 1). Patients aged ≥ 65 years had a significantly higher risk of complications following FD treatment (OR = 5.53; 95% CI: 1.79–17.06; *p* = 0.003) than those aged < 65 years (OR = 1.54; 95% CI: 0.56–4.24; *p* = 0.406), with the interaction effect approaching statistical significance (*P* for interaction = 0.097; Supplementary Figure 2).

## Discussion

Traditional endovascular coiling techniques for treating intracranial aneurysms carry the risk of intraoperative aneurysm rupture and hemorrhage. In contrast, FD, which involves the reconstruction of the parent artery lumen, effectively reduces the risk of rupture associated with manipulation inside the aneurysm and has recently become an important neurointerventional tool for treating intracranial aneurysms. Previous studies have demonstrated that FD is safe and effective in treating wide-neck intracranial aneurysms that are unsuitable for coil embolization. The results of this study demonstrate that FD and CET achieve comparable rates of complete occlusion for small- and medium-sized PSAs in economically developed regions of China. However, attention must be paid to the procedural safety of FD, and an extended follow-up is warranted to thoroughly assess its long-term efficacy in aneurysm occlusion.

Chalouhi et al. (24) reported higher occlusion and lower retreatment with FD versus coiling for aneurysms <10 mm. Di Maria et al. (25) likewise found greater occlusion with FD than coiling in 162 unruptured paraclinoid aneurysms (74.6% versus 49.1%; *p* = 0.005) despite shorter follow-up in the FD group (13.5 versus 31.5 months). In our cohort (median follow-up, 7 months), the occlusion rate in the FD group was 68.0%, which was slightly lower than that reported in previous studies. The occlusion rate was 66.7% in the coiling group, which was slightly higher than that in other related studies. Direct comparison by early occlusion is limited because coiling provides immediate neck closure but less durable long-term stability, whereas FD induces progressive thrombosis via hemodynamic modification, reducing intra-aneurysmal flow velocity, inflow, and wall shear stress, to mitigate late regrowth or rupture (26).

Adeeb et al. (27) found no significant difference in complete occlusion between the Pipeline Embolization Device (PED) and stent-assisted coiling for ophthalmic segment aneurysms (81.1% versus 75.9%). The PREMIER study (18) reported 83.3% complete occlusion at 3 years for small-to-medium aneurysms treated with FD. In our series, stent-assisted coiling achieved 97.2% complete occlusion, likely reflecting a smaller aneurysm size, a known predictor of recurrence risk (28–30). Stents may also enhance durability by reducing coil compaction and optimizing the parent artery angulation (31, 32). Although complete occlusion at the last follow-up differed between the groups, cumulative occlusion over time did not, consistent with slower FD occlusion kinetics and a relatively short follow-up period; longer-term, larger-scale studies are warranted.

Flow diversion improves aneurysm occlusion but raises concerns regarding procedural safety. In PARAT, Tubridge achieved higher occlusion than Enterprise stent-assisted coiling yet incurred more complications (8), a pattern echoed by meta-analyses showing increased procedure-related risks—including ischemia, hemorrhage, mortality, and visual dysfunction—despite better occlusion (OR = 1.4; *p* = 0.045) (20). In our cohort, the FD-related complication rate was consistent with that in prior reports (33), with stent malapposition as a principal driver (34, 35) that predisposes to acute thrombosis (36–38). Suboptimal apposition, particularly along the cervical segment, may delay endothelialization and sustain unfavorable hemodynamics in the aneurysm sac, contributing to incomplete occlusion (34). Accordingly, meticulous intraoperative assessment of deployment and apposition is essential, and balloon angioplasty or adjunctive stenting should be employed when apposition is inadequate (37). In-stent stenosis occurred in nearly 10% of FD cases, consistent with the literature, and likely reflects multifactorial contributors, such as smoking and intracranial atherosclerosis (39).

This study also found that age is an important interaction factor affecting the efficacy of the different treatment strategies. The occlusion advantage in the CET group was more pronounced in patients aged ≥ 65 years. The existing literature indicates that age is a significant factor that influences both the healing rate and occlusion effect in FD therapy (40, 41).

This study had some limitations. In interpreting our findings, several methodological factors collectively constrain causal inference. The retrospective, non-randomized design raises the possibility of selection and information bias and leaves room for residual confounding, despite mitigation measures. We predefined the selection criteria, captured relevant covariates as comprehensively as possible, and applied multivariable regression alongside PSM to reduce imbalance. A multicenter framework enhances representativeness but may introduce heterogeneity in operative techniques, imaging assessments, and perioperative management. Unified protocols and evaluation standards were implemented to limit inter-center variability; however, such variability cannot be fully eliminated.

These design constraints interact with limited follow-up in ways that affect time-dependent endpoints. With a mean follow-up of 12.6 months, variable visit intervals, and substantial attrition, the dataset cannot adequately capture the delayed and progressive occlusion characteristics of FD. Consequently, both the last-visit occlusion rate and cumulative occlusion probability for FD were likely underestimated, biasing comparisons against CET. Accordingly, a lack of short-term statistical differences does not preclude later divergence and precludes a robust evaluation of durability or rare but clinically meaningful events (e.g., ischemic complications, delayed rupture, and device issues). Therefore, these findings should be interpreted cautiously.

Although our cohort was relatively large for small-to-medium PSAs, non-random treatment allocation and marked group imbalance (CET: 595 versus FD: 93) introduced baseline selection bias. Differential and higher loss to follow-up (CET: 37.6% versus FD: 21.5%) further raises the risk of attrition bias. If patients with poorer prognoses are disproportionately lost, complete occlusion rates at the last follow-up may be overestimated, especially in the higher-attrition CET group, whereas cumulative complications may be underestimated. This informative censoring reduces confidence in the conclusions of “no significant difference,” particularly those derived from short-term survival analyses.

In summary, this study demonstrated that the overall occlusion rates between CET and FD for small- and medium-sized PSAs did not differ significantly in economically developed regions of China; however, the procedural safety of FD warrants careful evaluation. Subgroup analyses indicated that the advantages of CET were more evident in elderly patients. The retrospective design, multicenter heterogeneity, abbreviated and heterogeneous follow-up, group-size imbalance, and differential attrition indicate that comparative statements about the durability of aneurysm occlusion and device-related complications should be viewed as provisional. Future research should prioritize longer follow-ups with minimized attrition, standardized protocols with centralized imaging adjudication, and adequately powered prospective multicenter cohorts or randomized trials to validate and refine these findings.

## Data Availability

The original contributions presented in the study are included in the article/Supplementary material, further inquiries can be directed to the corresponding authors.

## Supplementary material

SUPPLEMENTARY FIGURE 1 The influence of the two treatment methods on the aneurysm occlusion rate in different patient subgroups. The benefit of CET was particularly pronounced (OR = 0.01; 95% CI: 0.00–0.08; *P* < 0.001) in patients aged ≥ 65 years, whereas the OR increased to 0.10 (95% CI: 0.04–0.24; *P* < 0.001) in patients aged < 65 years. The interaction effect of age stratification was statistically significant (*P* for interaction = 0.032). CET, conventional endovascular treatment; FD, flow diverters.

SUPPLEMENTARY FIGURE 2 The influence of the two treatment methods on the risk of all adverse events in different patient subgroups. Patients aged ≥ 65 years displayed a significantly higher risk of complications following FD treatment (OR = 5.53; 95% CI: 1.79–17.06; *P* = 0.003) than those aged < 65 years (OR = 1.54; 95% CI: 0.56–4.24; *P* = 0.406), with the interaction effect approaching statistical significance (*P* for interaction = 0.097). CET, conventional endovascular treatment; FD, flow diverters.
