# Supplementary figures and images for: Conventional endovascular treatment and flow diverter for unruptured small- and medium-sized paraophthalmic segment aneurysms
Source: Front Neurol. 2025 Nov 4;16:1648848. doi: 10.3389/fneur.2025.1648848 (PMC12623172; doi:10.3389/fneur.2025.1648848)

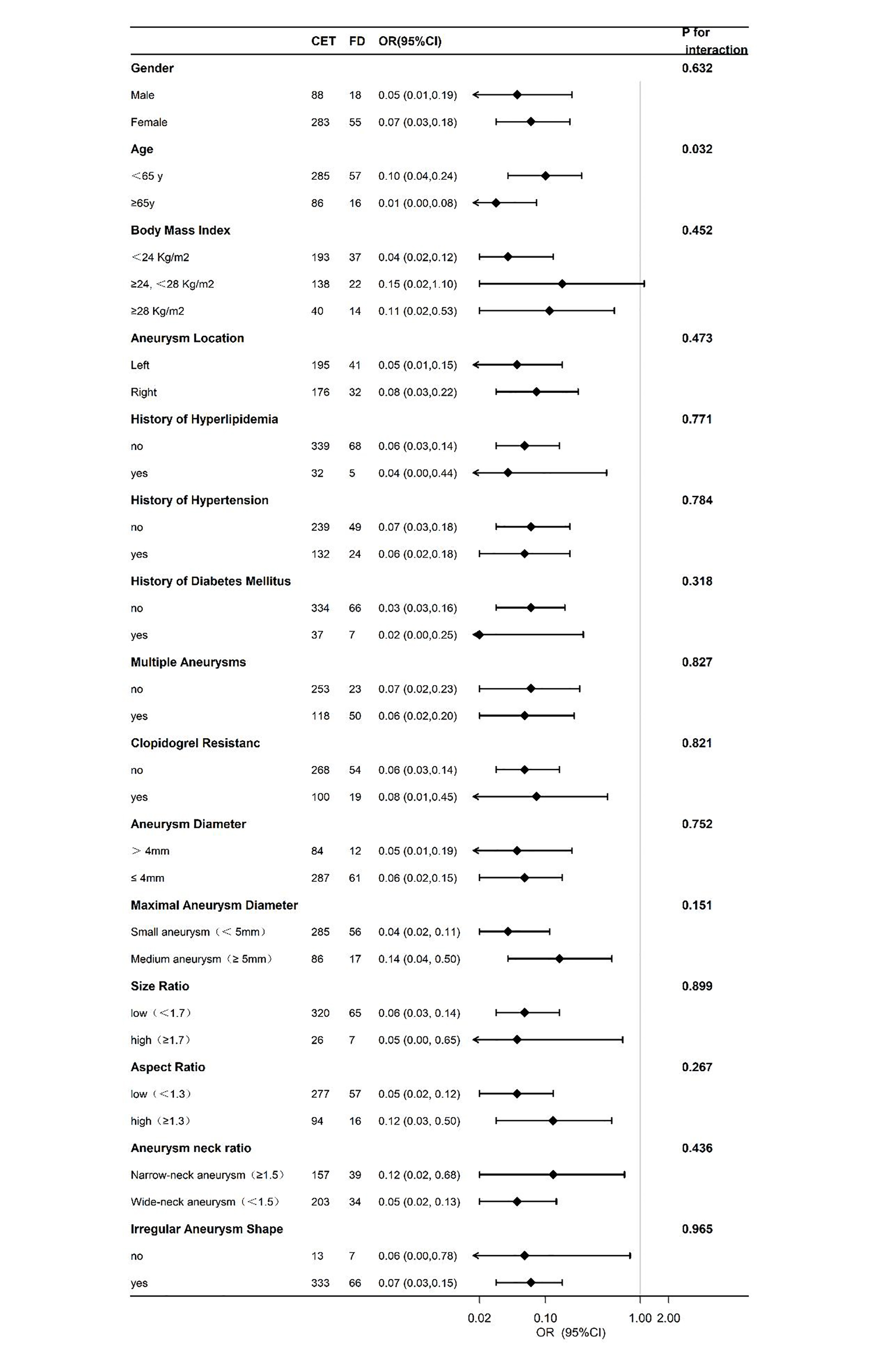

Supplement: SUPPLEMENTARY FIGURE 1 — The influence of the two treatment methods on the aneurysm occlusion rate in different patient subgroups. The benefit of CET was particularly pronounced (OR = 0.01; 95% CI: 0.00–0.08; P < 0.001) in patients aged ≥ 65 years, whereas the OR increased to 0.10 (95% CI: 0.04–0.24; P < 0.001) in patients aged < 65 years. The interaction effect of age stratification was statistically significant (P for interaction = 0.032). CET, conventional endovascular treatment; FD, flow diverters. [file Image_1.JPEG]

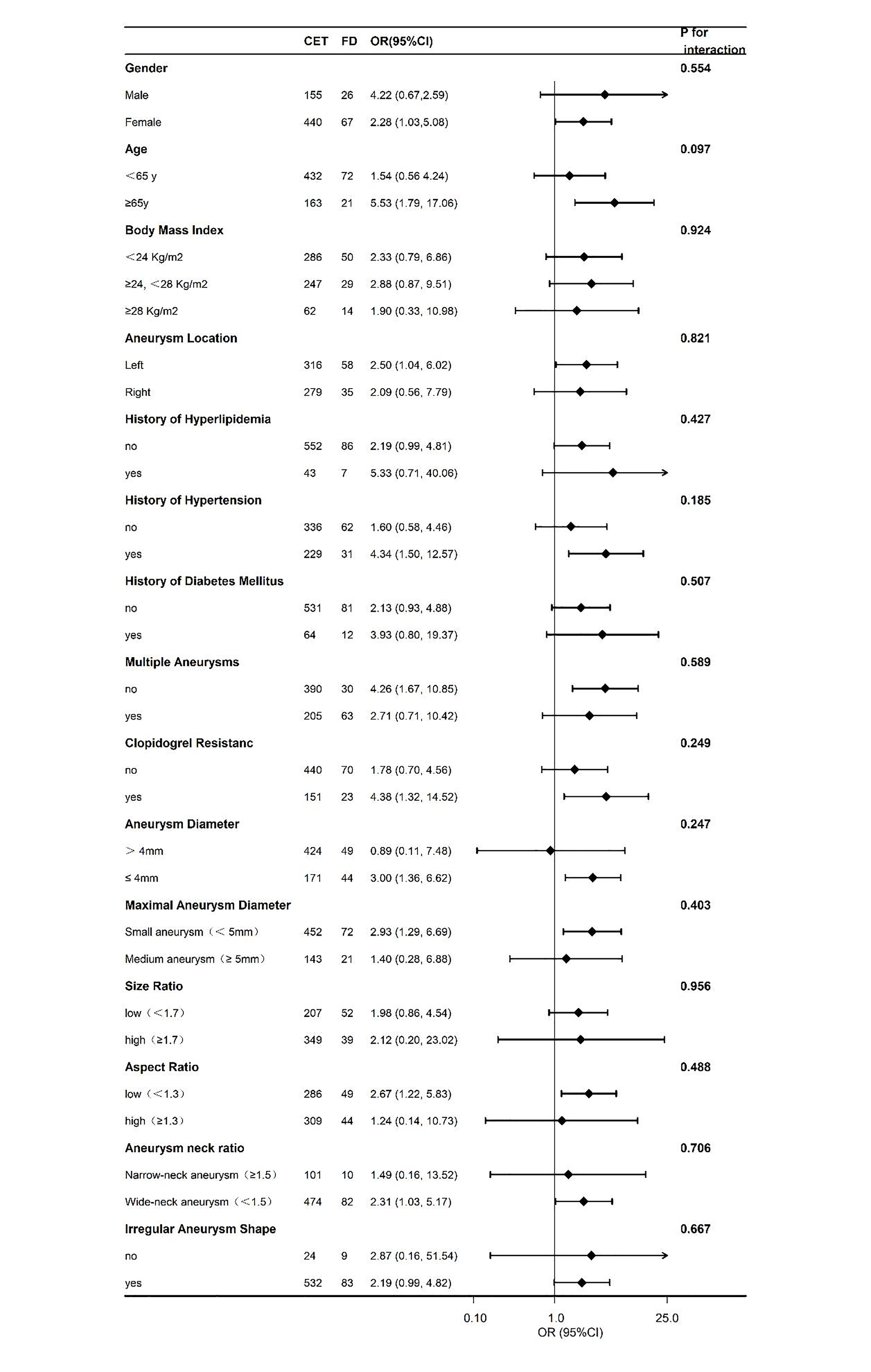

Supplement: SUPPLEMENTARY FIGURE 2 — The influence of the two treatment methods on the risk of all adverse events in different patient subgroups. Patients aged ≥ 65 years displayed a significantly higher risk of complications following FD treatment (OR = 5.53; 95% CI: 1.79–17.06; P = 0.003) than those aged < 65 years (OR = 1.54; 95% CI: 0.56–4.24; P = 0.406), with the interaction effect approaching statistical significance (P for interaction = 0.097). CET, conventional endovascular treatment; FD, flow diverters. [file Image_2.JPEG]
